# The deubiquitinase EIF3H promotes hepatocellular carcinoma progression by stabilizing OGT and inhibiting ferroptosis

**DOI:** 10.1186/s12964-023-01220-2

**Published:** 2023-08-09

**Authors:** Jianing Tang, Guo Long, Xuanxuan Li, Ledu Zhou, Yangying Zhou, Zheyu Wu

**Affiliations:** 1 Department of Liver Surgery, Xiangya Hospital, Central South University, Changsha, 410008 Hunan China; 2 National Clinical Research Center for Geriatric Disorders, Xiangya Hospital, Central South University, Changsha, 410008 Hunan China; 3 Department of Oncology, Xiangya Hospital, Central South University, Changsha, 410008 Hunan China; 4 Department of Orthopedics, The Second Affiliated Hospital of Naval Medical University, Shanghai, 200000 China

**Keywords:** Hepatocellular carcinoma, EIF3H, OGT, Ferroptosis, Deubiquitination

## Abstract

Hepatocellular carcinoma (HCC) is one of the most prevalent and lethal human malignancies, and with quite limited treatment alternatives. The proteasome is responsible for most of the protein degradation in eukaryotic cells and required for the maintenance of intracellular homeostasis. However, its potential role in HCC is largely unknown. In the current study, we identified eukaryotic translation initiation factor 3 subunit H (EIF3H), belonging to the JAB1/MPN/MOV34 (JAMM) superfamily, as a bona fide deubiquitylase of *O-*GlcNAc transferase (OGT) in HCC. We explored that EIF3H was positively associated with OGT in HCC and was related to the unfavorable prognosis. EIF3H could interact with, deubiquitylate, and stabilize OGT in a deubiquitylase-dependent manner. Specifically, EIF3H was associated with the GT domain of ERα via its JAB/MP domain, thus inhibiting the K48-linked ubiquitin chain on OGT. Besides, we demonstrated that the knockdown of EIF3H significantly reduced OGT protein expression, cell proliferation and invasion, and caused G1/S arrest of HCC. We also found that the deletion of EIF3H prompted ferroptosis in HCC cells. Finally, the effects of EIF3H depletion could be reversed by further OGT overexpression, implying that the OGT status is indispensable for EIF3H function in HCC carcinogenesis. In summary, our study described the oncogenic function of EIF3H and revealed an interesting post-translational mechanism between EIF3H, OGT, and ferroptosis in HCC. Targeting the EIF3H may be a promising approach in HCC.

Video Abstract

**Supplementary information:**

The online version contains supplementary material available at 10.1186/s12964-023-01220-2.

## Introduction

Hepatocellular carcinoma (HCC) is the most prevalent type of liver cancer (accounting for over 85% of all liver cancers globally), and it ranks the third in all cancer mortality and sixth in all cancer morbidity worldwide [1–3]. The current optimal therapeutic modality for HCC is radical surgical resection. However, due to lack of early symptoms and indications, a large number of patients present at an advanced stage and are unfit for surgery [2, 4]. Even with rapid improvement for other curative alternatives, such as chemotherapy, radiofrequency thermal ablation (RFTA), transcatheter arterial chemoembolization (TACE), molecular targeted therapy (sorafenib and Lenvatinib, etc.), as well as immune checkpoint inhibitors (ICIs), the prognosis is still dismal with 5-year overall survival rate of less than 20% [5–9]. Therefore, it is crucial to investigate the potential molecular mechanisms of the development of HCC to better improve the therapeutic responsiveness of HCC patients.

Ubiquitin–proteasome system (UPS) is the most prevalent protein degradation control system in eukaryotes. It regulates a variety of biological processes by degrading specific proteins and maintaining intracellular homeostasis [10, 11]. The protein ubiquitination is a vital post-translational modification and involves a cascade of cellular processes, including cell survival, DNA repair, cell cycle progression, apoptosis, and antigen presentation [12, 13]. Ubiquitination regulation is a reversible process, generally mediated by deubiquitinating enzymes (DUBs), which can effectively cleave ubiquitin (Ub) from substrates to enhance its stability and maintain the dynamic balance of the ubiquitination process [14]. Up to now, more than 100 kinds of DUBs have been discovered and can be classified into six different families: ubiquitin-specific proteases (USP), the JAB1/MPN/MOV34 family (JAMM), Ubiquitin COOH-terminal hydrolases (UCH), ovarian tumor proteases (OTU), motif interacting with ubiquitin-containing novel DUB family (MINDY), and Josephins [15]. Eukaryotic translation initiation factor 3 subunit H (EIF3H), belonging to the JAMM family of DUBs, is recognized as the largest eukaryotic translation initiation factor [16, 17]. In addition to its general function in initiating translation, the amplification and elevation of EIF3H have been observed in various malignancies, such as breast cancer, lung cancer, prostate cancer, esophageal carcinoma, and also HCC [18–22]. Besides, overexpression of EIF3H has also been related to the oncogenesis and drug sensitivity [23, 24]. However, it is unknown how it functions mechanistically as a deubiquitinase in HCC.

*O-*GlcNAcylation affects serine and/or threonine residues of cytoplasmic, nuclear, and mitochondrial proteins, and is a widespread dynamic and reversible post-translational modification (PTM) [25, 26]. This monosaccharide alteration has been identified as a crucial regulator of numerous significant biological and pathological processes, including signal transduction, protein interactions, and enzymatic activity [27]. Unlike most other PTMs, *O-*GlcNAcylation is catalyzed by only two conserved enzymes, the *O-*GlcNAc transferase (OGT) and *O-*GlcNAcase (OGA), in the addition and removal of *O-*GlcNAc, respectively [28]. Previous studies revealed that OGT may contribute to the tumorigenesis of several malignancies. The elevated *O-*GlcNAcylation and OGT expression have been involved in facilitating tumor growth and metastasis in HCC [29, 30], and the repression of OGT may be a promising treatment modality for nonalcoholic fatty liver disease [31]. However, the potential mechanisms regarding OGT activation or overexpression in HCC have not yet been explored.

Ferroptosis is a form of intracellular iron-dependent cell death that differs from autophagy, necroptosis, and apoptosis morphologically, genetically, and biochemically [32, 33]. Ferroptotic cell death is caused by oxidative damage induced by lipid peroxidation and intracellular iron-induced reactive oxygen species (ROS) accumulation, which is associated with glutathione (GSH) depletion and glutathione peroxidase 4 (GPX4) inactivation, and an eventual iron-dependent lipid peroxide accumulation [34]. At present, increasing studies have demonstrated that ferroptosis has a role in regulating tumor growth, and acted as an effective therapeutic approach for cancer treatments [35, 36]. Several studies have also reported that the regulatory proteins of ferroptosis, such as P53, GPX4, and solute carrier family 7 member 11 (SLC7A11) play a critical role in the development of hepatocarcinoma [37–39]. However, it is unknown whether other potential proteins could regulate ferroptosis and participate in the pathogenesis of HCC.

In this study, we conducted an unbiased screening of 10 JAMM family DUBs from a DUBs siRNA library and explored that EIF3H was the most potent DUB for decreasing the level of *O*-GlcNAcylation and OGT. EIF3H may work as a deubiquitinase response for OGT deubiquitinating and stabilization in HCC. Moreover, we also observed that EIF3H promoted cell proliferation and invasion by regulating ferroptosis. Therefore, our findings have demonstrated that EIF3H is a promising deubiquitinating enzyme of OGT and participated in the progression of HCC through ferroptosis, which provides new insights into clinical diagnosis and drug target exploration of HCC.

## Materials and methods

### Cell culture

The human liver cancer cell lines LM3, Hep3B, and human embryonic kidney HEK293 cells were acquired from American Type Culture Collection (ATCC). All cells were maintained in Dulbecco’s Modified Eagle’s Medium (DMEM, 41,965, Life Technologies) with 10% fetal bovine serum (FBS). They were all cultured in a humidified incubator at 37 °C and in an atmosphere of 5% CO_2_.

### Plasmids and RNA inference

The wild-type and mutant constructs of EIF3H plasmids were purchased from the Hanbio Biotechnology Co., Ltd. (Shanghai, China). The HA-K48, HA-K63, and HA-Ub plasmids were acquired from Addgene. The small interfering RNAs targeting EIF3H (5ʹ-GCAACTCTTGGAAGAAATATA-3ʹ) and (5ʹ-CCCAAGGATCTCTCTCACTAA-3ʹ) were gained from Ruibo Biotechnology Co., Ltd. (Guangzhou, China). Lipofectamine 2000 (Invitrogen, Carlsbad, CA, USA) was utilized for plasmid and siRNA transfection according to the manufacturer's recommendations.

### Cell proliferation analysis

The cell proliferation analysis was performed using EdU incorporation assay, Cell Counting Kit-8 (CCK8) assay, and clone formation assay. The EdU incorporation assay was performed using Cell-LightTM EdU Apollo 567 In Vitro Kit (RiboBio, Guangzhou, China) followed by the manufacturer’s instruction. The results were analyzed by Image-Pro Plus 6.0 software (Media Cybernetics, USA). For the CCK8 assay, cells were seeded at 1 × 10^3^ cells/well into the 96-well culture plates. The 10 μl CCK8 solution reagent was added into each well and incubated for 2 h at 37 °C. Then, a microplate reader was used to detect the absorbance at 450 nm. For the colony formation, cells were trypsinized and seeded into six-well plates at a density of 1,000 cells/well. After fourteen days of incubation, cells were fixed with 4% paraformaldehyde and stained with 0.5% crystal violet. All experiments were conducted three times with three replicates individually.

### Cell migrasion analysis

We utilized the 8 μm pore polycarbonate membranes transwell chambers (Corning, USA) for transwell migration assay. About 5 × 10^5^ cells were suspended in a serum-free medium and were implanted into the Matrigel-coated top chambers. Then, the bottom chambers were added with 600 μl complete medium. The cells on the bottom side of the chamber were fixed and stained with crystal violet after 24 h.

### Western blot analysis

RIPA extraction reagent (Beyotime, China) combined with protease inhibitors was used to lyse cells (Sigma-Aldrich, USA). Proteins were separated on a 10–12.5% of sodium dodecyl sulfate–polyacrylamide gel electrophoresis (SDS-PAGE), and transferred onto a polyvinylidene difluoride membrane (PVDF, Millipore, USA). Primary antibodies were EIF3H (Proteintech, 11,310–1-AP), OGT (Proteintech, 66,823–1-Ig), O-GlcNAc (Santa cruz, sc-59623), Myc (Proteintech, 60,003–2-Ig), HA (Proteintech, 51,064–2-AP), and GAPDH (Proteintech, 60,004–1-Ig). Chemiluminescent detection was carried out utilizing an enhanced chemiluminescence (ECL) kit (Boster, China).

### Immunofluorescence assay

LM3 and Hep3B cells were fixed with 4% paraformaldehyde for 30 min at room temperature, and permeabilized for 5 min with 0.2% Triton X-100. Cells were then blocked for 1 h with 10% goat serum. Primary antibodies against EIF3H (rat, Proteintech, China) and OGT (mouse, Proteintech, China) were incubated overnight at 4 °C. After that, Fluorochrome-conjugated secondary antibodies were applied to respond to the primary antibodies in the dark and then incubated with DAPI at room temperature. A NIKON80i fluorescence microscope was used to capture images.

### Protein stability assays

To measure the half-live of OGT, LM3 and Hep3B cells were seeded in 24-well plates and transfected with siEIF3H or siControl. After 24 h, cells were treated with 100 µM protein synthesis inhibitor cycloheximide (Sigma-Aldrich) for indicated time points. Western blot was performed to detect protein levels.

### Co-immunoprecipitation (Co-IP) assay

Co-IP lysis buffer (Beyotime, China) was applied to lyse the cells. Cell lysis was precleared with rabbit IgG for 2 h, and the specified antibody was then immunoprecipitated at 4 °C overnight. The lysates were then mixed with Protein A/G PLUS-Agarose beads (Santa Cruz, USA) and incubated at 4 °C for 2 h. The immunocomplexes were separated by SDS-PAGE after washing three times with lysis buffer and followed by a standard immunoblotting process.

### Xenograft tumor model

Four-week-old BALB/c nude female mice were acquired from Beijing HFK Bioscience Co., Ltd. in China for the xenograft tumor model. Mice were randomly assigned to two groups (*n* = 6 per group). 1 × 10^6^ LM3 cells were injected into each mouse, and tumor sizes were measured every five days. Then, the tumors were weighed and photographed when the mice were sacrificed after inoculation for fifty days. The experimental animal facility at Xiangya Hospital of Central South University kept the mice in a pathogen-free, temperature- and humidity-controlled environment. The ethics committee at Xiangya Hospital of Central South University authorized the animal protocols.

### In vivo deubiquitination assay

In vivo deubiquitination analysis was carried out in HCC and HEK293T cells. For in vivo deubiquitination assay, HA-Ub, Flag-OGT, GFP-EIF3H plasmids were transfected into HEK293T cells for 48 h. Cells were then treated with 10 μM MG132 (MCE) for 6 h. Then cells were washed with pre-chilled phosphate-buffered saline (PBS) and lysed with RIPA extraction reagent (supplementary with 1% SDS). HA-ubiquitinated OGT was isolated using an anti-Flag antibody. The ubiquitination level of OGT was detected by Western blotting with an anti-HA antibody.

### Measurement of ROS assay

For total ROS detection, cells were collected and trypsinized into single cells, and were resuspended in RPMI-1640 medium with 10% FBS and 400 μM DHE (Sigma, USA) probe after being cleaned with 1xPBS. The samples were then incubated in a dark room for 15 min at 37 °C with 5% CO_2_. Following a wash with preheated PBS, cells were examined using flow cytometry (Fortessa, USA). Similarly, for lipid ROS determination, cells were gathered and resuspended in an RPMI-1640 medium containing 10% FBS before being treated with 10 M C11-BODIPY (Thermo Fisher, USA). The samples were also incubated for 30 min at 37 °C and 5% CO_2_ and shielded from light. The samples were washed with PBS and then being examined by flow cytometry (Fortessa, USA).

### Iron assay

To measure the amount of iron in each cell line, the total iron and ferrous iron (Fe^2+^) were detected by using the Iron Assay Kit (Sigma-Aldrich; USA). We suspended five volumes of iron assay buffer to release the iron. For total iron detection, we added 5 μL iron reducer for each sample well and blank control well. For ferrous iron detection, we added 5 μL iron assay for each sample well and blank control well. The sample plates were incubated for 30 min at room temperature with shake and dark. Following that, each well was mixed with 100μL of the iron probe, and incubated for 1 h at room temperature with shake and shielded from light. Finally, the absorbance was measured at 593 nm using a TECAN sunrise spectrophotometer (Tecan, Switzerland).

### Statistical analysis

To compare two and more groups, the Student's t-test and one-way ANOVA were utilized, respectively. The Bonferroni adjustment was applied for multiple comparisons. All tests had a two-tailed design and a *p*-value < 0.05 were regarded as statistically significant. All statistical tests were carried out by using Prism 7.0 (GraphPad, USA).

## Results

### EIF3H depletion inhibits *O*-GlcNac pathway activity in HCC

**Fig. 1:**
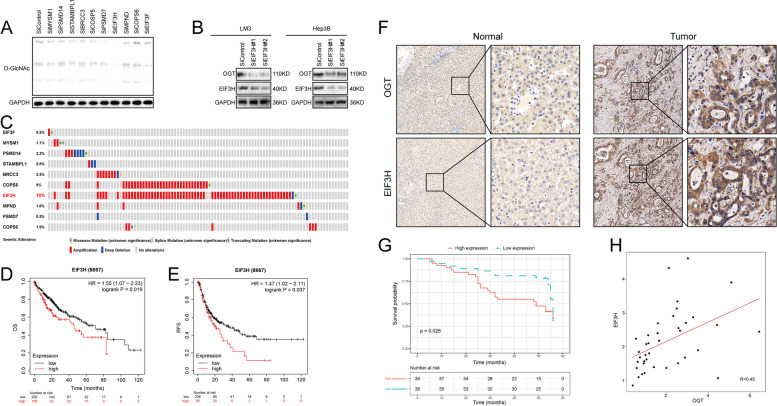
EIF3H depletion decreases *O*-GlcNac pathway activity and correlates with poor prognosis in hepatocellular carcinoma.** A** The siRNAs specific to each deubiquitinating enzyme (JAMMs family) were transfected into LM3 cells. After 48 h, cells were lysed and the *O*-GlcNac and OGT protein levels were analyzed by Western blot. **B** EIF3H depletion reduced OGT protein levels in both LM3 and HepG2 cells. **C** Genetic alternations of JAMMs family DUBs in HCC. The OncoPrint schematic was constructed in cBioPortal (TCGA dataset). **D-E** Prognostic analysis between *EIF3H* expression and OS **(D)** and RFS (**E**) in TCGA HCC patients. **F** Immunohistochemistry analysis of EIF3H and OGT expression in the Xiangya HCC cohort (*n* = 97), including normal liver tissue (*n* = 20) and HCC tumor tissues (*n* = 77). **G** Kaplan–Meier curve analysis of the EIF3H in Xiangya HCC cohort. **H** Correlations between EIF3H and OGT protein levels in Xiangya HCC cohort (Pearson correlation, *R* = 0.45). HCC, hepatocellular carcinoma; DUBs, deubiquitinating enzymes; OS, overall survival; RFS, relapse-free survival

To investigate the potential role of JAMMs family DUBs in the involvement of the *O*-GlcNac signaling pathway in HCC, we transfected ten different siRNA mixtures specific for each JAMMs deubiquitinating enzyme into LM3 cells. We discovered that EIF3H depletion caused a decrease in total *O*-GlcNac levels (Fig. 1A). By employing two nonoverlapping siRNAs targeting EIF3H, we further explored that EIF3H depletion decreased the OGT protein levels in both LM3 and Hep3B cells (Fig. 1B). Genetic analysis from cBioPortal database revealed that among all the JAMMs in human HCC, EIF3H had the most genetic alterations, and gene amplifications were observed in about 18% of all cases (Fig. 1C).

### EIF3H correlates with OGT and leads to poor prognosis in HCC

We further performed a survival analysis of the EIF3H using the TCGA dataset and found that high expression of EIF3H was associated with poor prognosis in HCC patients, with both decreased inferior overall survival (OS) and relapse-free survival (RFS) (Fig. 1D, E). Consistently, we performed an immunohistochemistry analysis of tissue microarrays from Xiangya hospital, which showed that OGT was positively associated with EIF3H, and that high expression of EIF3H was associated with unfavorable prognosis (Fig. 1F-H).

### EIF3H interacts with OGT in HCC

**Fig. 2:**
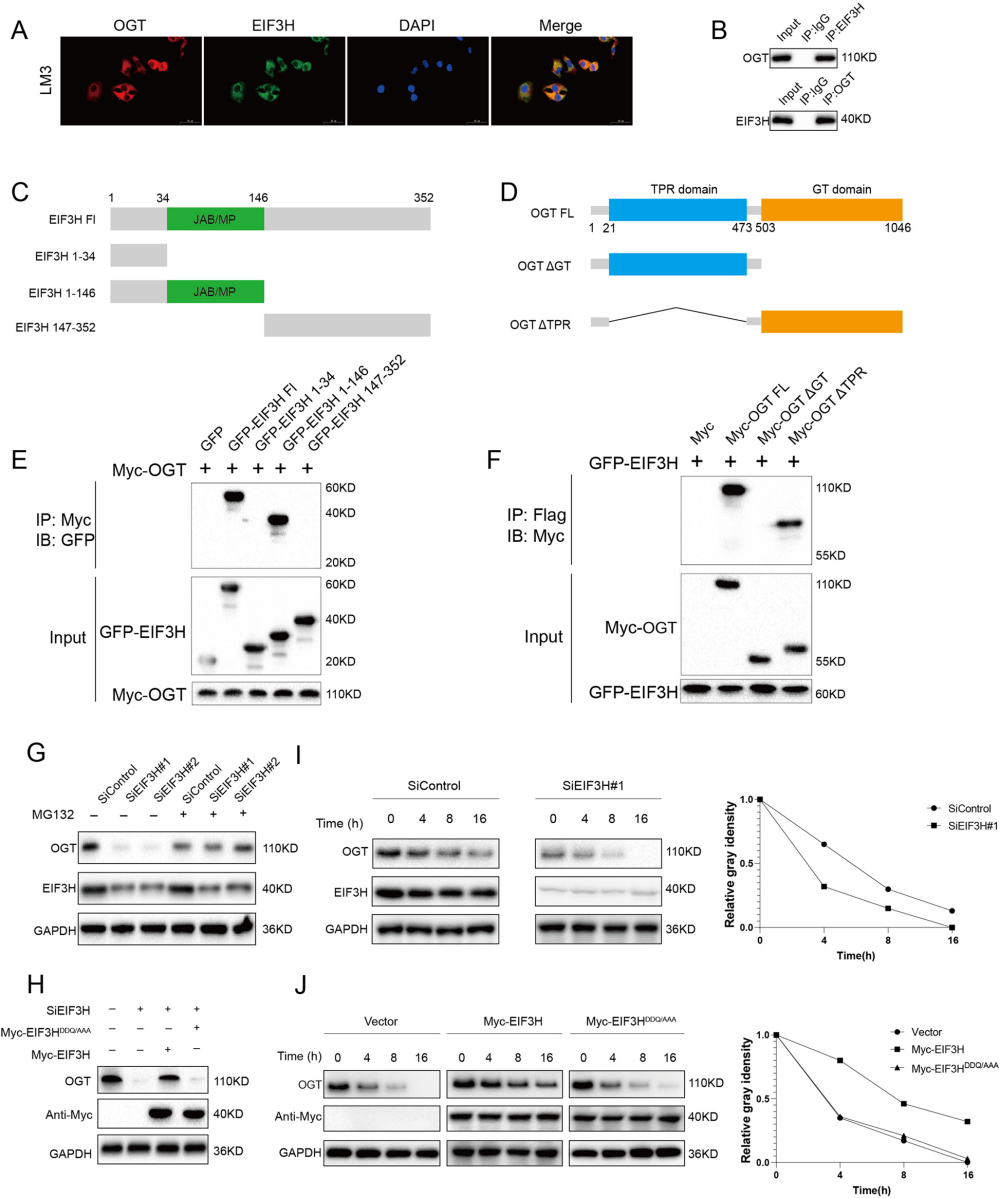
EIF3H associates with OGT and enhances its stability. **A** The immunofluorescence assay presented that EIF3H and OGT at least partially colocalized in LM3 cells.** B** Co-IP assay showed a correlation between endogenous EIF3H and OGT in LM3 cells. **C-D** EIF3H and OGT structure domain and deletion mutants were applied in the study. **E** The JAM/MP domain of EIF3H interacted with OGT. HEK293 cells were transfected with 2 µg Myc-OGT and GFP-EIF3H full-length or mutants. Cells were harvested with NP-40 lysis buffer for 24 h. Co-IP was performed using a GFP antibody and the possible interacted EIF3H domains were detected by Myc antibody. **F** OGT interacted with EIF3H through its GT domain. HEK293 cells were transfected with 2 µg GFP-EIF3H together with Myc-OGT full-length or mutants., Cells were harvested with NP-40 lysis buffer After 24 h. Co-IP was performed using Myc antibody. The possible interacted OGT domains were detected by GFP antibody. **G** In the presence of the proteasome inhibitor MG132, the knockdown of EIF3H did not further reduce the OGT protein expression level. LM3 cells were transfected with siControl or siEIF3H. After 48 h, cells were treated with 10 µM MG132/vehicle for 6 h, cell lysates were prepared for Western Blot analysis. **H** LM3 cells were transfected with EIF3H (wild type or mutant DDQ/AAA) together with EIF3H siRNA. The OGT protein levels were detected by western blot assay. **I** Silencing EIF3H decreases OGT half-life in LM3 cells. LM3 cells were transfected with siControl or siEIF3H. After 48 h, cells were treated with 100 µM cycloheximide/vehicle for indicated times. Cell lysates were prepared for western blot analysis. **J** The mutant EIF3H^DDQ/AAA^ did not increase OGT half-life in HEK293 cells. HEK293 cells were transfected with Myc-tag, Myc-EIF3H or Myc-EIF3H^DDQ/AAA^ plasmids. After 24 h, cells were treated with 100 µM cycloheximide/vehicle for indicated times. Cell lysates were prepared for Western blot assay

We further carried out an immunofluorescence assay to determine the cellular localization of EIF3H and OGT. The results showed that EIF3H and OGT at least partially co-localized in HCC cells **(**Fig. 2A**)**. Furthermore, co-IP data showed that endogenous EIF3H coimmunoprecipitates endogenous OGT in LM3 cells, indicating a functional relationship between EIF3H and OGT **(**Fig. 2B**)**. We then generated a number of truncated OGT and EIF3H constructs and demonstrated that the JAB/MP domain of the EIF3H interacted with the GT domain of OGT **(**Fig. 2C-F**)**.

### EIF3H enhances the stability of OGT in HCC

We further assessed whether OGT was deubiquitinated by EIF3H. We observed that the depletion of EIF3H by siRNA decreased the OGT protein level apparently (Fig. 2G). While the proteasome inhibitor MG132 or overexpression of EIF3H-WT could reverse this effect, but not the catalytic inactive mutant (Fig. 2H). To further prove EIF3H could affect the stability of OGT, we then treated LM3 cells with the protein synthesis inhibitor cycloheximide (CHX). In cells depleted of EIF3H, the half-life of OGT was decreased (Fig. 2I). On the other hand, in cells overexpressing wild type EIF3H, but not the catalytically inactive mutant EIF3H^DDQ/AAA^, had a higher half-life of OGT (Fig. 2J). These findings demonstrated that EIF3H increased the stability of OGT in a DUB-dependent manner.

### EIF3H deubiquitylates OGT in HCC

**Fig. 3:**
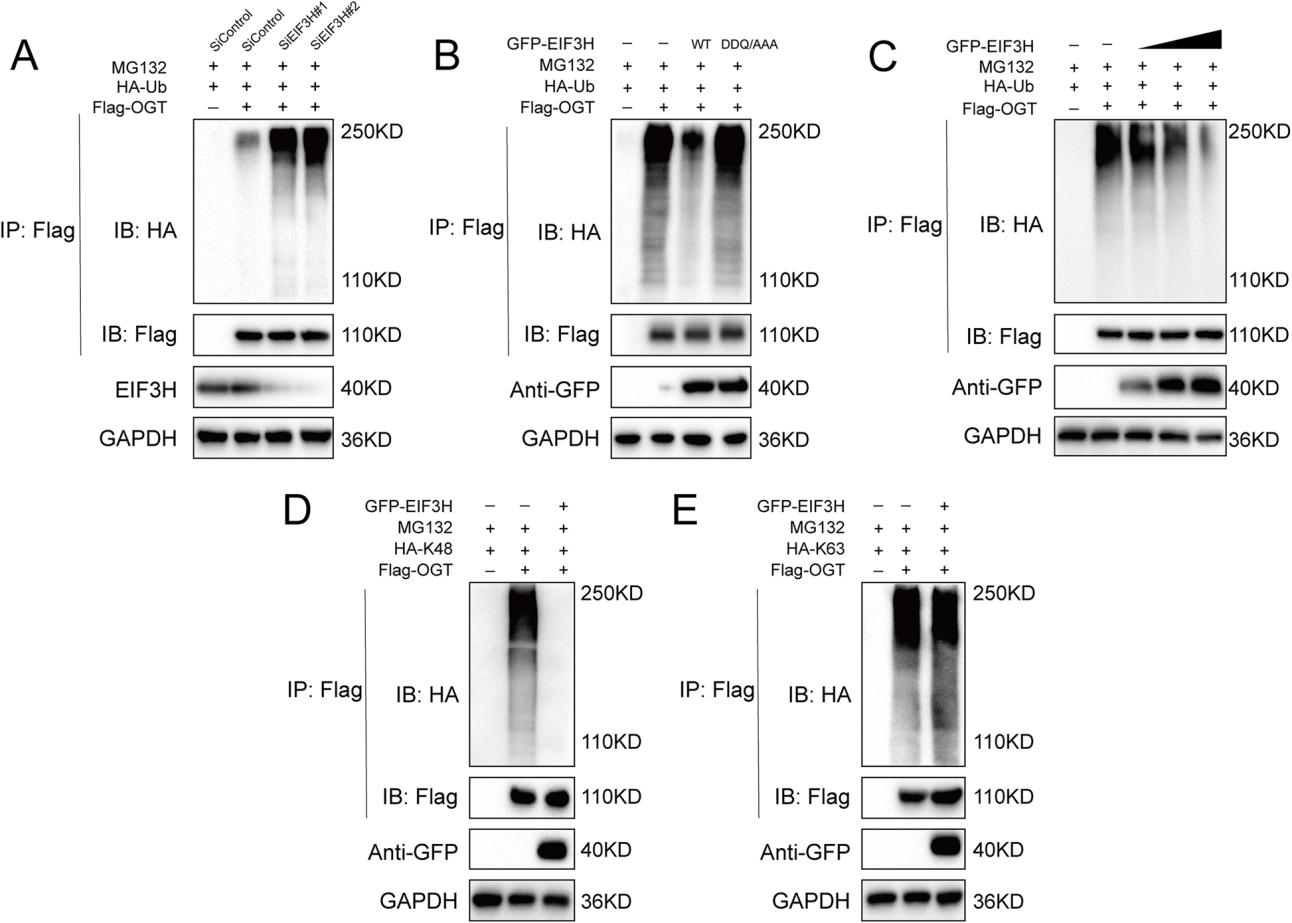
EIF3H deubiquitylates OGT.** A** OGT was immunoprecipitated with anti-OGT and immunoblotted with anti-HA. LM3 cells transfected with the indicated siEIF3H were treated with MG132 for 6 h before collection. **B** Immunoblotting to detect the ubiquitination of OGT in HEK293 cells co-transfected with Flag-OGT, HA-Ubiquitin and GFP-EIF3H (wild type or DDQ/AAA). **C** EIF3H removed the ubiquitin chain of OGT in a dose-dependent manner. **D-E** K48 or K63 Ub was co-transfected with Flag-OGT and GFP-EIF3H into HEK293 cells. After treatment with 10 μM MG132 for 6 h, cell lysates were subjected to ubiquitination assay and the ubiquitination level of OGT was detected by HA antibody

As EIF3H is a member of the JAMM family of deubiquitylase, we continued to determine the possibility that EIF3H deubiquitylates OGT. As illustrated in Fig. 3A, we observed that the depletion of EIF3H significantly elevated the level of ubiquitinated-OGT in LM3 cells. Inversely, ectopic expression of EIF3H-WT, but not its EIF3H^DDQ/AAA^, markedly reduced OGT ubiquitylation in cells (Fig. 3B). We further discovered that EIF3H deubiquitylated OGT in a dose-dependent manner through in vitro deubiquitylation assays (Fig. 3C). In addition, we investigated EIF3H deubiquitinating activity on OGT on two of the most common ubiquitination sites (lysine (K) 48-linked and lysine (K) 63-linked ubiquitination). To investigate which type of ubiquitin chains on OGT were affected by EIF3H, we co-transfected cells with OGT, EIF3H, HA-tagged WT ubiquitin and the two most common ubiquitin chains (K48-specific and K63-specific ubiquitin). The results demonstrated that EIF3H could efficiently remove K48-linked ubiquitin chains on OGT (Fig. 3D, E). Collectively, EIF3H was regarded as a specific DUB to deubiquitinize and stabilize OGT.

### EIF3H promotes HCC progression in vitro and in vivo

**Fig. 4:**
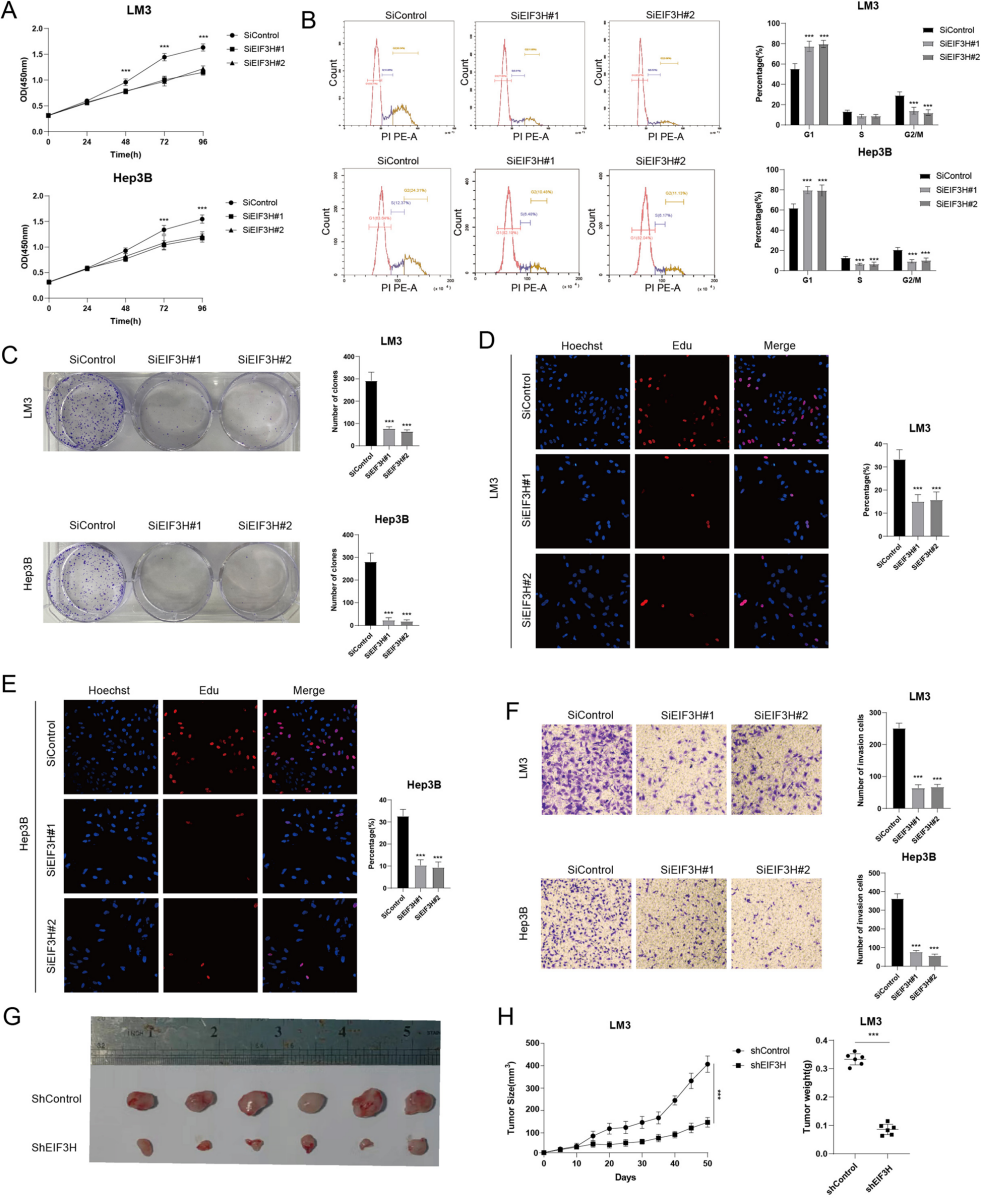
EIF3H depletion inhibits HCC proliferation and migration. **A** EIF3H repression inhibited cell proliferation in HCC cells. **B** Knockdown of EIF3H-induced G1 cell cycle arrest in HCC cells. **C** Silence of EIF3H decreased clone formation capability of HCC cells. **D-E** Representative images of EdU assays of LM3 and Hep3B cells. **F** Transwell invasion assay of HCC cells. **G**-**H** EIF3H depletion inhibits tumor growth in vivo. LM3 cells were stably transfected with lentivirus carrying a scrambled shRNA or EIF3H shRNA. 1 × 10^6^ LM3 cells were injected into each mouse (*n* = 6). Tumor sizes were measured every five days until the end of the experiment. ***P value < 0.001

We further explored the biological functions of EIF3H in HCC cells (LM3 and Hep3B). The results demonstrated that the depletion of EIF3H decreased cell proliferation dramatically (Fig. 4A). Depletion of EIF3H increased the proportion of cells in G1 phase of the cell cycle, implying that EIF3H may participate in the regulation of G1 to S transition in HCC cells (Fig. 4B). Colony formation assay also showed that EIF3H depletion led to a reduction in cell survival (Fig. 4C)**.** Consistently, EdU incorporation assay revealed that knockdown of EIF3H inhibited the DNA synthesis in LM3 and Hep3B cell lines (Fig. 4D, E)**.** Moreover, transwell assays showed that the depletion of EIF3H decreased cell migration capacity (Fig. 4F). In addition, we further utilized xenograft mice models to investigate the role of EIF3H in the regulation of tumor growth in vivo. The results illustrated that EIF3H depletion by lentivirus-based shRNA suppressed the HCC tumor growth markedly (Fig. 4G, H)**.**

### EIF3H regulates ferroptosis in HCC cells

**Fig. 5:**
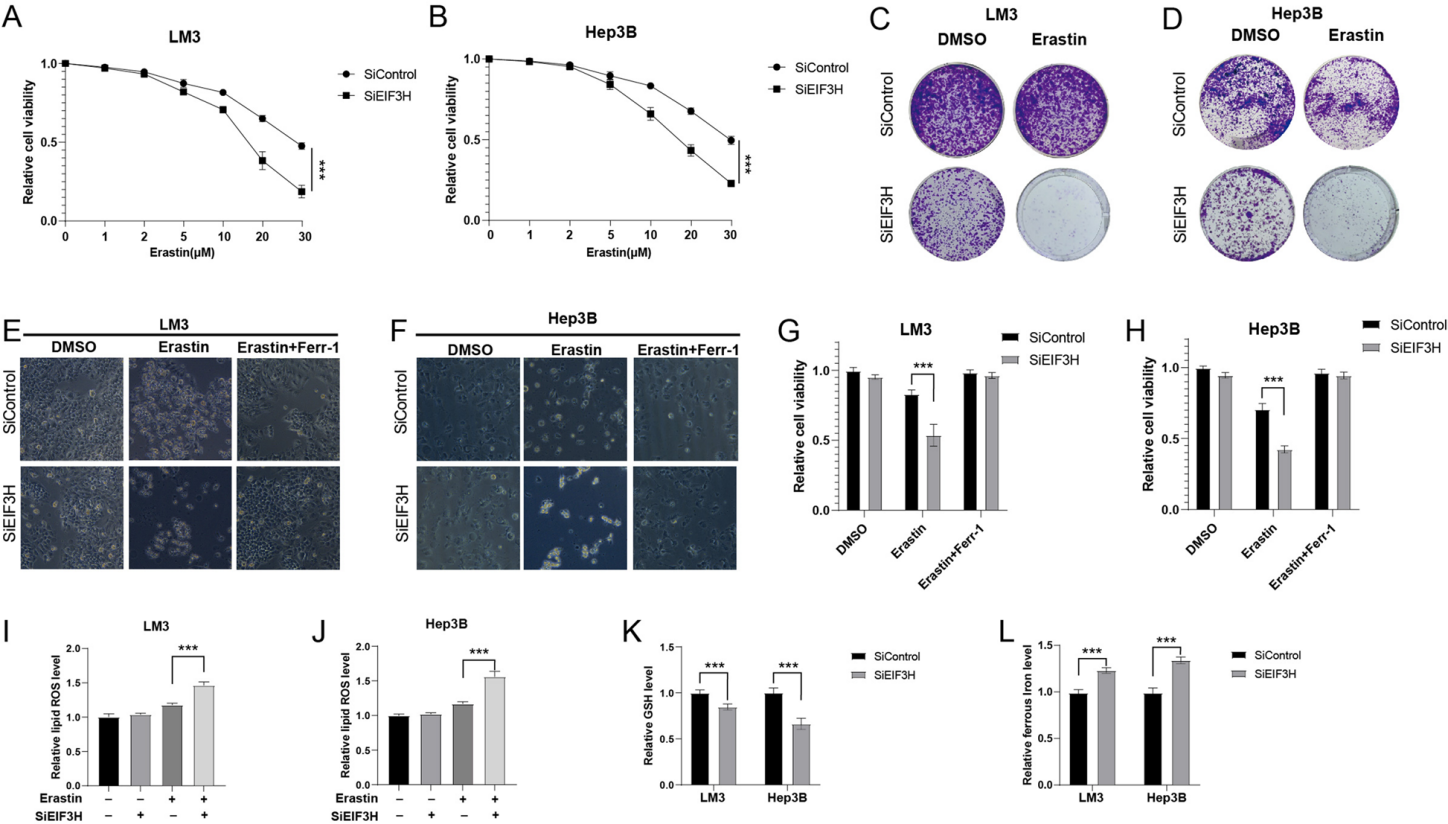
EIF3H regulates ferroptosis in HCC cells. **A**-**B** Cell viability assay showing the response of LM3 (**A**) and Hep3B (**B**) cell lines to different concentrations of Erastin with or without *EIF3H* depletion. **C-D** Colony formation showing the response of LM3 (**C**) and Hep3B (**D**) cell lines to Erastin with or without *EIF3H* depletion. **E–F** Representative images illustrating LM3 (**E**) and Hep3B (**F**) cells response to Erastin (20 μM) ± Ferrostatin (1 μM) with or without *EIF3H* knockdown. **I-K** Levels of lipid ROS (**I**, **J**), GSH (**K**), and ferrous iron (**L**) in HCC cells with or without *EIF3H* depletion. ***P value < 0.001

To better explore the biological role of EIF3H in HCC development, we further investigate the effect of EIF3H on ferroptosis. We observed that the depletion of EIF3H decreased cell proliferation rate (Fig. 5A, B) and clonogenic potential (Fig. 5C, D) in both LM3 and Hep3B cell lines in response to different concentrations of Erastin. We then intended to characterize the role of EIF3H in ferroptosis by evaluating the erastin-induced ferroptosis with or without ferrostatin, an inhibitor of ferroptosis. Knockdown of EIF3H increased erastin-induced growth inhibition of HCC cells **(**Fig. 5E-H). Moreover, we also found that the knockdown of EIF3H increased the intracellular concentration of lipid ROS (Fig. 5I, J), ferrous iron (Fig. 5L**)**, and decreased Glutathione (GSH) (Fig. 5K). Taken together, our results indicate that the deletion of EIF3H promoted the ferroptosis in HCC cells.

### EIF3H regulates HCC cell proliferation and migration through OGT and ferroptosis

**Fig. 6:**
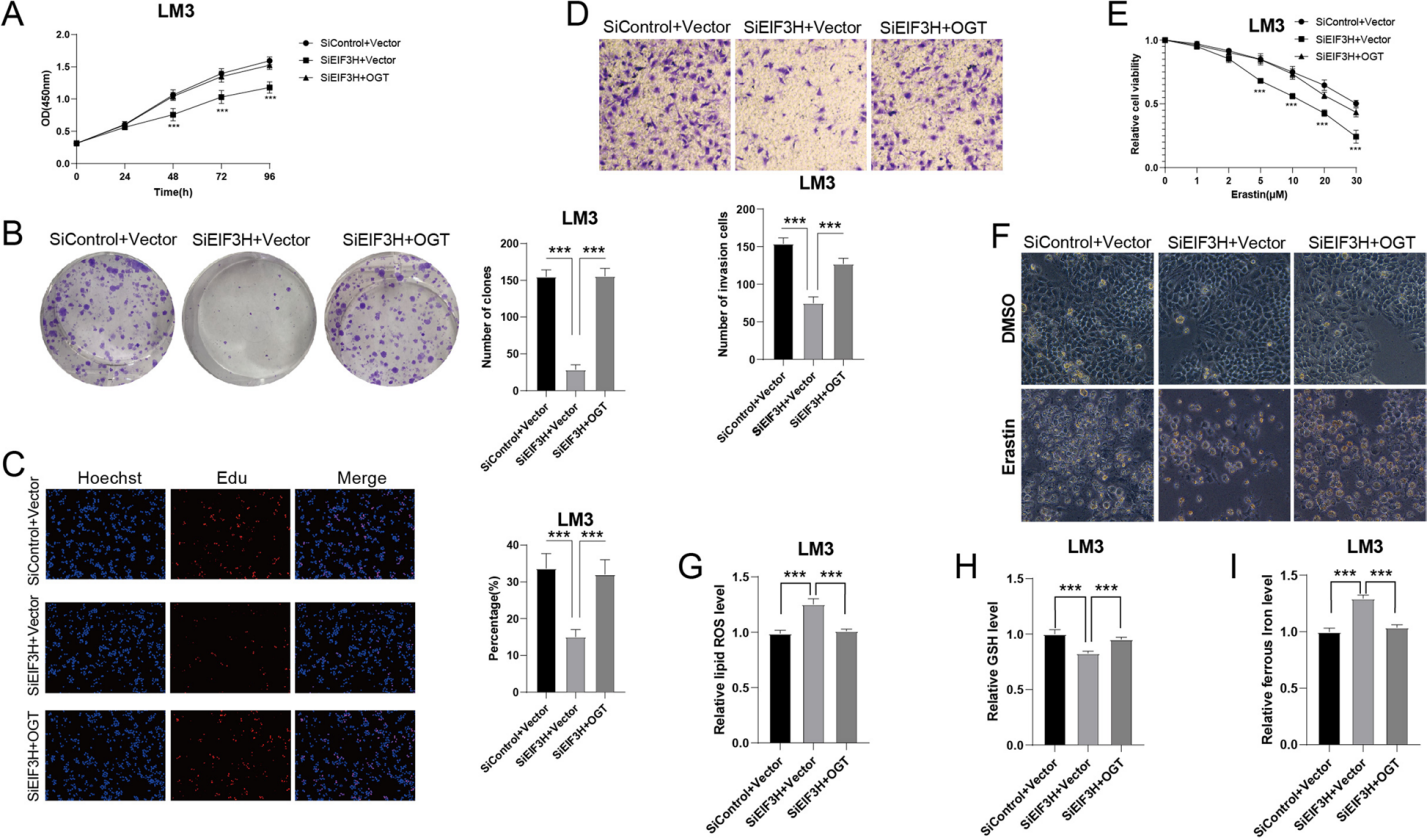
Increased OGT expression reverses the effect of EIF3H depletion. **A** Cell proliferation assay of LM3. **B** Colony formation of LM3. **C** Representative images of EdU assay of LM3. **D** Transwell invasion assay of LM3. **E** Cell Viability analysis showing the response of *EIF3H*-knockdown LM3 cells to different concentrations of Erastin with or without *OGT* overexpression. **F** Representative images presenting *EIF3H*- silenced LM3 cells response to Erastin (20 μM) with or without *OGT* overexpression. **G**-**I** Levels of lipid ROS (**G**), GSH (**H**), and ferrous iron (**I**) in *EIF3H*-depleted LM3 cells with or without *OGT* overexpression. ***P value < 0.001

To determine the functions of EIF3H in regulating HCC cell progression by stabilizing OGT and regulating ferroptosis, we further performed rescue experiments. By overexpressing OGT in EIF3H-knockdown cells, the CCK8 assay demonstrated that ectopic expression of OGT markedly reversed the growth inhibition induced by EIF3H repression (Fig. 6A). Overexpression of OGT also rescued the colony formation ability of LM3 cells (Fig. 6B). Coincidentally, the increased OGT facilitated DNA synthesis in EIF3H-depleted LM3 cells (Fig. 6C). Moreover, transwell assays showed that the suppressive function induced by EIF3H ablation was largely restored by OGT overexpression (Fig. 6D). These results suggested that EIF3H promoted hepatocellular carcinoma cell proliferation via the regulation of OGT. In addition, we further explored whether EIF3H inhibits the HCC cell ferroptosis through OGT. By overexpressing OGT in EIF3H-depleted LM3 cells, we discovered that OGT elevation rescued cells from EIF3H-dependent ferroptosis (Fig. 6E, F). Consistently, the elevated intracellular concentration of lipid ROS and ferrous iron, and reduced GSH can also be reversed by OGT expression (Fig. 6G-I). These data indicated that OGT expression can suppress ferroptosis promoted by EIF3H in HCC cells. Taken together, these results confirmed that EIF3H promoted OGT expression and inhibited ferroptosis in HCC cells.

## Discussion

As one of the most frequently diagnosed and life-threatening human malignancies in the world, the available treatment alternatives are quite limited for HCC [2]. Moreover, the high heterogeneity of HCC and numerous risk factors make prognosis prediction considerably more challenging, and the overall prognosis remains poor [40]. Thus, it is critical to investigate the potential pathogenesis that takes part in the onset and progression of HCC. Protein ubiquitination is a vital post-translational modification for cellular protein degradation and homeostasis maintenance [41]. The regulation of ubiquitination is a reversible process that is constantly mediated by different E3 ubiquitin ligases and DUBs [42]. Accumulating evidence has showed that DUBs play an indispensable role in the occurrence and progression of cancers [43–47]. However, the potential roles of DUBs in HCC are largely unknown.

EIF3H, a DUB belonging to the JAMM superfamily, is recognized as the largest eukaryotic translation initiation factor and is associated with malignant phenotypes in multiple cancers [16, 17]. However, little is known about the enzymatic role of EIF3H in HCC. Besides, previous studies have demonstrated that OGT may contribute to the tumorigenesis of several malignancies, and it has been reported that elevated *O-*GlcNAcylation and OGT expression were involved in the facilitating of tumor growth and metastasis in HCC [29, 30]. However, the potential mechanisms regarding OGT activation, or whether OGT is regulated by DUBs in HCC, have not yet been explored. In the present study, we discovered EIF3H as a modulator of OGT deubiquitination and stabilization in HCC. Depletion of EIF3H significantly reduced OGT protein levels and inhibited *O*-GlcNac signaling activity. We observed that EIF3H had the most genetic instability among all the JAMMs and was overexpressed in HCC. Tissue microarray data showed a correlation between EIF3H and OGT expression levels. Furthermore, survival analysis indicated that high expression of EIF3H was related to a worse clinical outcome in HCC. We then investigated the underlying molecular mechanism of EIF3H in regulating OGT. Firstly, EIF3H and OGT interacted with each other directly. Endogenous EIF3H and OGT were co-immunoprecipitated in LM3 cells, suggesting that the interaction between EIF3H and OGT is physiological. Binding experiments revealed that EIF3H interacted with GT domain of OGT independent of its DUB activity. Secondly, EIF3H improved OGT protein stabilization in a DUB activity-dependent manner. We found that the knockdown of EIF3H significantly repressed OGT protein levels. Intriguingly, the reduced OGT can be recovered by overexpressing wild-type EIF3H, but not its catalytically inactive mutant, which indicated that the catalytic activity of EIF3H may have influenced the stability of OGT. We then treated cells by CHX to inhibit protein synthesis, and discovered that EIF3H depletion reduced OGT half-life, whereas overexpression of wild-type EIF3H prolonged it. We further analyzed the linkage of ubiquitin chains in interaction between EIF3H and OGT. We observed that EIF3H could efficiently remove the K48-linked ubiquitin chains on OGT. Previous studies revealed that K48-linked ubiquitination frequently led to proteasomal degradation [48, 49] [54, 55], so the results suggested that EIF3H may sustain the OGT stability by eliminating the K48-linked ubiquitin chain from the OGT protein. Finally, EIF3H could promote cancer progression in HCC via OGT. Our results showed that the depletion of EIF3H markedly decreased the proliferation and migration of HCC both in vitro and in vivo. Moreover, the suppression effects induced by EIF3H repression could be restored by the ectopic expression of OGT. Taken together, these results confirmed that EIF3H accelerated the proliferation and migration through increasing OGT stability.

Ferroptosis is a new non-apoptotic programmed cell death triggered by lipid ROS and has been regarded as a potential therapeutic target for cancer therapy. Several clinical and preclinical drugs, including sorafenib and Erastin could induce ferroptosis and have anticancer properties in HCC [38]. In the current study, we demonstrated that the depletion of EIF3H increased the intracellular concentration of lipid ROS, ferrous iron concentration, and decreased GSH, indicating its inhibitory role in ferroptosis. In addition, we also confirmed that EIF3H promoted HCC cell proliferation and migration via upregulation of OGT. Meanwhile, OGT expression can suppress ferroptosis promoted by EIF3H in HCC. Correspondingly, our findings are the first to reveal that EIF3H inhibits ferroptosis by interacting with OGT, thereby accelerating HCC cell growth and tumor progression.

In conclusion, our study investigated the biological functions of EIF3H in HCC, and we revealed that EIF3H is a post-translational modulator in regulating OGT stabilization and deubiquitination. Moreover, we also demonstrated that EIF3H may accelerate the HCC progression via the regulation of OGT expression and ferroptosis. Therefore, EIF3H could be a promising therapeutic target for the treatment of HCC.

### Supplementary information

**Additional file 1. **The original, uncropped gels or blots in the study.

## Data Availability

The data that support the findings of this study are available from the corresponding author upon reasonable request.
