# Supplementary figures and images for: The deubiquitinase EIF3H promotes hepatocellular carcinoma progression by stabilizing OGT and inhibiting ferroptosis
Source: Cell Commun Signal. 2023 Aug 9;21:198. doi: 10.1186/s12964-023-01220-2 (PMC10413709; doi:10.1186/s12964-023-01220-2)

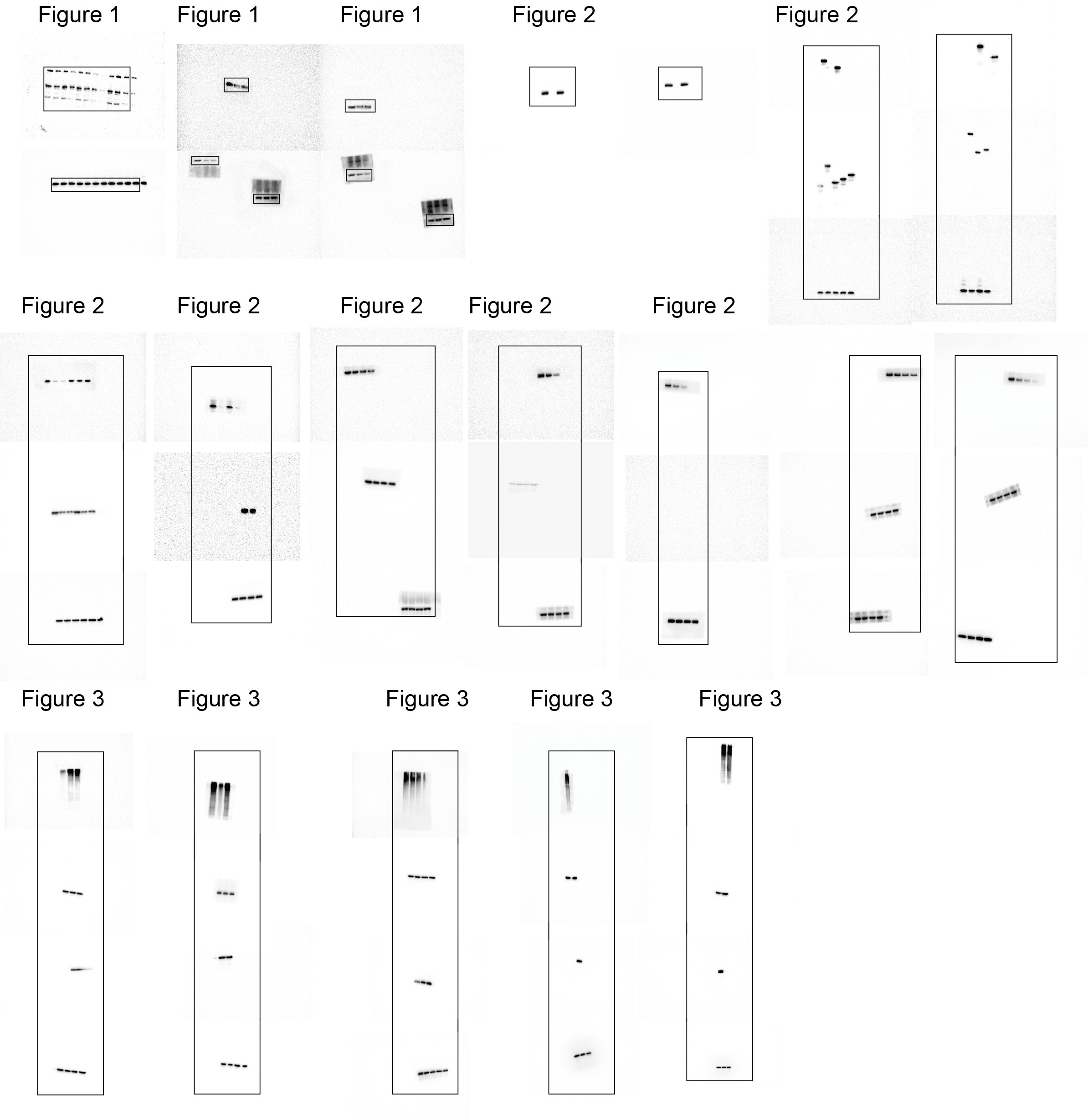

Supplement: Supplementary file 2 — Additional file 1. The original, uncropped gels or blots in the study. [file 12964_2023_1220_MOESM1_ESM.docx]
